# The involvement of the hypothalamo-pituitary-adrenocortical axis in stress physiology and its significance in the assessment of animal welfare in cattle

**DOI:** 10.4102/ojvr.v84i1.1398

**Published:** 2017-04-28

**Authors:** Emma J. Brown, Andre Vosloo

**Affiliations:** 1 School of Life Sciences, University of KwaZulu-Natal, Westville Campus, South Africa

## Abstract

The intensification of cattle production has raised concern for animal welfare due to the stress that is associated with farming practices. The welfare of an animal is determined by the animal’s ability to cope with or adapt to its continuously changing environment and the biological cost that is associated with this adaptation and maintenance. Stressors arise from various psychological, physiological and physical aspects of farming practices due to management and human–cattle interactions. Measuring the activity of the hypothalamo-pituitary-adrenocortical (HPA) axis with plasma cortisol levels is a useful method for determining the effects of stress on animals as it is stimulated at the onset of a perceived stress. The activation of the HPA axis affects various target tissues or systems and can result in suppression of the immune system, increased susceptibility to disease and adverse effects on reproductive success in prenatal and neonatal calves. Although some levels of stress associated with farming practices are unavoidable, improvements in farming methods need to be implemented in order to maintain or increase the efficiency of cattle production in a way that does not compromise the welfare of the animal.

## Introduction

Cattle are considered the predominant farm animals that are required for the production of meat and milk for human consumption (Phillips 2010). Despite the obvious value of cattle for providing 18% of protein intake and 9% of energy intake in the human diet (Phillips 2010), the recent intensification of cattle production has led to an increase in concern for animal welfare regarding management and human–cattle interaction (Lynch 2010). Members of the public and animal rights groups have placed cattle farming practices under great scrutiny, declaring that animal experimentation and intensive animal agriculture have led to insufficient attention to animal welfare (Dohms & Metz 1991).

When formulating a definition for the term animal welfare, Lynch (2010) states that this term cannot simply be explained by one definition. The U.K. Farm Animal Welfare Council formulated the five ‘freedoms’, which serve as a guideline when constructing a definition for the term animal welfare (Lynch 2010). The five ‘freedoms’ are: (1) freedom from thirst, hunger and malnutrition, (2) freedom from discomfort, (3) freedom from pain, injury and disease, (4) freedom to express normal behaviour and finally (5) freedom from fear and distress. Despite these guidelines, there is still great ambiguity that surrounds the term ‘welfare’. However, universal definitions that do exist have various determinants that encompass three main classes: (1) the production capacity of the animal, (2) the physiological function of the animal and (3) the feelings of the animal. An example of a universally accepted definition of the term welfare is the state of the animal in relation to its immediate environment and that the state of health, prosperity and well-being is attained by the ability of the animal to respond to external stimuli. The term ‘state’ refers to the feelings of the animal and the diverse physiological and behavioural responses as well as their general health (Lynch 2010).

In the livestock industry, it is economically essential to maintain high reproductive efficiency. To ensure optimal production is maintained, a balance needs to exist between increased production and the elimination of undesirable impacts of environmental stressors. In order to establish this balance, knowledge of how stress affects animals is essential (Dreiling, Carman & Brown 1991).

The perception of stress from either an internal or external stimulus (Burdick et al. 2011) by an animal results in an abnormal or severe adjustment in its physiology (Lynch 2010). The stressor, therefore, poses a threat to, and disrupts homeostasis (Burdick et al. 2011) which is defined as the coordination of physiological processes that sustain a dependable state in an organism (Chen et al. 2015). Due to the fact that an animal’s immediate environment is not static and is subject to continuous unpredictable changes (Möstl & Palme 2002), all life forms must respond to these environmental changes (Dohms & Metz 1991) by stimulating a behavioural, autonomic, neuroendocrine or immunological response (Von Borell, Dobson & Prunier 2007).

These responses are evoked due to the fact that the perception of stress directly stimulates the hypothalamo-pituitary-adrenocortical (HPA) axis (Lay et al. 1996), which results in the release of stress-related hormones (Burdick et al. 2011) in an effort to regulate homeostasis (Manteca, Mainau & Temple 2013). Activation of the HPA axis in response to stress (Lay et al. 1996) enables the animal to cope with external or internal stress stimuli (Lynch 2010). Stress can be defined as either ‘good’ stress known as eustress or ‘bad’ stress known as distress (Le Fevre, Matheny & Kolt 2003). Eustress is described as a positive stress and may have a beneficial effect on the animal (Antoniou & Cooper 2005). Distress occurs when the demands that are placed on the body (both physiological and psychological) surpass the body’s capacity to expend energy in order to maintain homeostasis (Le Fevre et al. 2003). Eustress is proposed not to be inherently bad for the animal (Antoniou & Cooper 2005; Moberg 2000); however, overstimulation or under stimulation of the coping mechanism due to prolonged distress (Le Fevre et al. 2003; Moberg 2000) can ultimately render the animal vulnerable to disease and failure to reproduce and develop properly (Moberg 2000). Increased disease susceptibility and failure to reproduce therefore indicate that the animal has difficulty coping (Broom 1991), which poses a threat to the well-being of the animal (Moberg 2000) and in turn, the welfare of the animal (Broom 1991). It is therefore essential that one is able to differentiate between non-threatening eustress and distress that alters the biological state of the animal, resulting in adverse consequences for its welfare (Moberg 2000).

This review, therefore, serves to evaluate the physiology of stress, the effects that stress has on immune function and reproductive success and how this relates to the welfare and productivity of the animal. Firstly, the evaluation must take into account the types of stress that cattle are subjected to and how these stressors may be classified. The involvement and role of the HPA axis must also be considered and mention must be given to the hormones that are secreted and the components that are activated. The review will then address the factors that contribute to how an animal perceives and reacts to a stress and the consequences that stress has on immune function and reproductive success. Finally, a brief evaluation of the significance of the HPA axis in determining the biological functioning, and hence the welfare of the animal, will be given.

## The classification and types of stress

### Origin

Cattle experience various stressors throughout the production cycle (Carroll & Forsberg 2007) which may arise from endogenous and exogenous sources (Table 1).

**TABLE 1:** The exogenous and endogenous stressors that affect cattle.

Endogenous	Exogenous
Genetic or physical state:	Social environment:
• Breed	• Housing
• Sex	• Feeding
• Temperament	• Stocking density
• Behaviour	• Spatial allowance
• Weight	
Social state:	Physical environment:
• Competition	• Temperature
• Aggression	• Humidity
• Leadership	• Wind
• Dominance	

*Source*: Adapted from Lynch, E.M., 2010, *Characterisation of physiological and immune-related biomarkers of weaning stress in beef cattle*, Doctoral dissertation, Department of Biology and National Institute for Cellular Biotechnology, National University of Ireland Maynooth

### Perception

Whether endo- or exogenous in origin, stressors are perceived by the animal as either psychological, physical or physiological stress (Carroll & Forsberg 2007). As cattle usually experience a combination of stressor groups (Lynch 2010), the cumulative stress response may have detrimental effects on the welfare of the animal (Manteca et al. 2013).

### Duration

Stress can be classified as chronic or acute, depending on the duration for which the animal is subjected to a particular stress. *Acute stress* arises when an animal experiences a stressor for a short period of time and can be associated with the fight or flight response (Hughes et al. 2013). Acute stress is involved in preparing the immune system to stimulate adaptation for a short period of time (Hughes et al. 2013). *Chronic stress* is a result of long-term exposure to a stressor resulting in a prolonged disruption to the homeostatic state. The stress response shifts from preparing the immune system to suppressing the immune system. The transition between acute and chronic stress is dependent on the intensity of the psychological perception of the animal to a particular stressor. The duration for which the stressor triggers a stress response and the ability of the animal to overcome a stressful event are influenced by previous exposure, genetics, sex and the temperament of the animal (Hughes et al. 2013).

Over the last century, our understanding of the physiology and behaviour of cattle has improved. A better understanding of the intricate regulatory processes, complex social structure and the highly developed learning ability of cattle has prompted the re-evaluation of the effect that farming practices and conditions have on both the effectiveness of production and the welfare of the animal (Lynch 2010). Lynch (2010) highlighted six areas of animal production that bring about stress to the animal and therefore may affect animal welfare: (1) the ill treatment and physical abuse of an animal, (2) neglect, by accident or ignorance, (3) inadequate design in accommodation and housing, leading to insufficient space, unsuitable floor types and poor feed and water access, (4) inadequate management and poor husbandry practices, (5) poorly executed mutilations, such as tail dockings, dehorning and castration and (6) poor condition of procedures such as transport and practices at the market and slaughter house.

Direct indications of harm to the welfare of cattle are reduced productivity and increased mortality (Lynch 2010). There are, however, early warning approaches to measure and investigate the effects of stress (De Kloet et al. 2005) due to the central control mechanisms of the HPA axis (Lay et al. 1996). These early, direct and unbiased measurements of the biological state of the animal are vital for attempts to assess animal welfare and bring about improvements in housing and management of cattle in the production industry (Broom 1991).

## The hypothalamo-pituitary-adrenocortical axis

The HPA axis can be regarded as a crucial neuroendocrine system that is involved in the control of diverse physiological processes and adaptations to stress (Mormède et al. 2007). When the environmental pressure of a perceived stress exceeds that to which an animal’s adaptive mechanisms can accomodate (Kumar, Manuja & Aich 2012), this system produces energetic metabolites that arise either from energy storage tissues or from the transformation of proteins into energetic metabolites (Mormède et al. 2007). The energy provided by this process is then used to fuel a behavioural, autonomic, neuroendocrine or immunological response (Von Borell et al. 2007) to help the animal cope with the stressor (Mormède et al. 2007). The body systems that are paramount to this process of adaption to a perceived stress (Kumar et al. 2012) are found within the central nervous system. The sympathetic-adrenomedullary (SAM) axis and the HPA axis aid in the process of linking the initial perception of the stress to an adequate response (Lynch 2010).

For the purpose of this review, emphasis will be placed on the mechanisms of the HPA axis, due to the fact that it is involved in the regulation of bodily processes in response to long-term, chronic stress (Kumar et al. 2012).

There are many steps involved in the initial perception of a stress and the stimulation of an adequate response that will bring about the regulation of homeostasis (Chen et al. 2015). Moberg and Mench (2000) outlined the biological model known as the general adaptation syndrome that animals have developed in order to cope with a perceived stress.

**FIGURE 1:**
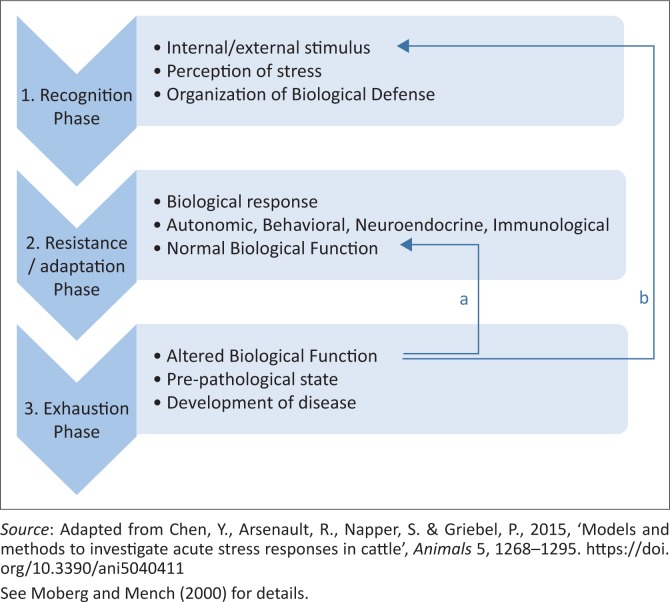
The stages involved in the biological general adaptation syndrome in response to stress in animals.

The perception of stress requires a change in the biological function of the animal in order to cope with and reduce the negative effects associated with a stress response (Figure 1b). The achievement of regaining homeostasis results in the normal biological function of the animal being restored (Figure 1a) (Lynch 2010).

**FIGURE 2:**
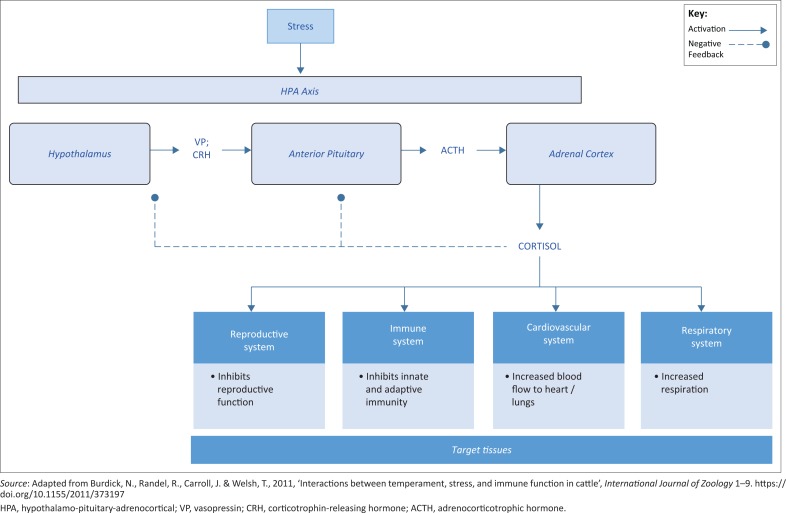
The response of the hypothalamo-pituitary-adrenocortical axis to stress.

Due to the fact that the SAM is activated in response to a short-term or acute stress, its inability to rectify a stressful event results in the activation of the HPA axis (Lynch 2010), which, as mentioned earlier, is involved in resolving long-term, chronic stress (Kumar et al. 2012). Figure 2 illustrates the HPA axis pathway, associated hormones and target systems.

Cortisol, a primary glucocorticoid in cattle, is released from the adrenal cortex and distributed via the circulatory system to various target tissues or organs or systems in the body (Figure 2) (Burdick et al. 2011). In order for glucocorticoids to be transported via blood in the circulatory system, carrier proteins must be present (Burdick et al. 2011). Albumin is considered to be the main cortisol binding globulin (Burdick et al. 2011). The severity of the effect that glucocorticoids exert on the target organs or tissues or systems is dependent upon six factors: (1) the amount of hormone that is secreted, (2) the duration of hormone secretion, (3) the peripheral blood concentration and cortisol binding globulins, (4) the abundance of glucocorticoid receptors in target tissues, (5) the tissue on which they exert an effect and (6) the extent of the breakdown of glucocorticoid metabolites (Burdick et al. 2011).

Due to the fact that glucocorticoids are the final effectors of the HPA axis, they play a vital role in the control of homeostasis and the basal cortisol concentrations (Lynch 2010). Glucocorticoids also play a role in the mechanism of negative feedback (Burdick et al. 2011). When the hypothalamus and anterior pituitary detect high concentrations of cortisol, the release of vasopressin (VP) and corticotrophin-releasing hormone (CRH) from the hypothalamus and adrenocorticotrophic hormone (ACTH) from the anterior pituitary is inhibited, resulting in inhibition of the synthesis of cortisol from the adrenal cortex (Burdick et al. 2011) and termination of the stress response (Lynch 2010).

Although the HPA axis can be seen as advantageous in the restoration of the homeostasis to its normal state, failure to terminate the stress response can result in the overstimulation and dysregulation of the homeostatic system, resulting in a phenomenon known as allostatic load or overload (Beerde 1997; Lynch 2010). Termination failure may be a result of stimulation and activation of an inadequate response to the perceived stressor or continuous habituation to the stimulus is not attained (Lynch 2010). Ultimately, the consequences of prolonged over- or under-activity of the allostatic system are detrimental to immune function (Beerde 1997) and the reproductive success of the animal (Kumar et al. 2012), which in turn raises questions regarding its welfare (Broom 1991).

## Interactions between the genetic and physical state of cattle on the hypothalamo-pituitary-adrenocortical axis activity and stress perception

The activity of the endocrine system, incorporating the HPA axis, is commonly used as an indicator of stress in animals (Lynch 2010). Although this method provides an accurate evaluation of animal welfare, one should be aware that the function of the HPA axis is highly heritable (Mormede et al. 2011) and therefore results in differences both between different breeds of cattle and within various breeds of cattle (Grandin 1997). This individual variation gives rise to differences in HPA axis activity (Mormede et al. 2011) and generalised stress responses. Individual variation is seen to be a result of the genetic and physical state of cattle and includes the breed, sex, age, temperament and behaviour of the species (Lynch 2010).

Temperament can be defined as the reactivity of cattle to humans and their immediate environment. There are many factors that contribute to whether an animal may perceive a situation as being stressful, which include developmental history, prior experience and genetic factors. These factors contribute to whether the induced stress response is beneficial or harmful to the animal (Burdick et al. 2011).

Temperament is influenced by genetics and is a hereditable trait which can affect an animal’s response to handling (Grandin 1997). Highly domesticated animals that are accustomed to routine handling have shown to have subtle responses to changes in the environment and human interaction, whereas wilder species have shown an increasingly elevated response to environmental changes and handling procedures (Grandin 1997).

As a result of more temperamental animals causing greater injury to themselves and to cattle handlers (Burdick et al. 2011), cattle used in production industry are selected based on docility in order to improve animal welfare, performance and human safety (Norris et al. 2014). Wilder species of cattle are also seen to have higher basal cortisol concentrations which impact growth rates, reproduction and weakens immune responses to pathogens (Grandin 1997).

## Stress and immune function

When an environmental or internal stress stimulus is perceived, it is paramount that the stress response is prompt, efficient and regulated in order to alleviate increased susceptibility to pathogens. Stress affects the mechanisms of innate and adaptive immunity (Lynch 2010), and although these systems are not mutually exclusive (Salak-Johnson & McGlone 2007), there is a complex interaction of communication between the two (Lynch 2010). Innate immunity refers to the ancient evolutionary mechanism that is evoked immediately or several hours (0–4 h) after the perception of an antigen. Innate immunity includes the body’s physical barriers such as the skin and mucous membranes as well as complement and antigen non-specific cellular components. Innate immunity is non-specific and the body’s first-line defence to a perceived pathogen (Carroll & Forsberg 2007). When functioning optimally, pathogens that are encountered on a daily basis are prevented from causing disease as their invasion is blocked by the body’s physical barriers (Carroll & Forsberg 2007). Effector cells of the innate immune system such as macrophages, dendritic cells and B-cells, also known as professional antigen presenting cells, possess pattern recognition receptors that subsequently recognise the pathogen-associated molecular pattern and trigger the effector cells to perform their required function (Medzhitov & Janeway 2000). The pattern recognition receptors aid in detecting and eliminating the pathogens from the body (Carroll & Forsberg 2007) and account for the prompt kinetics of the innate immune response (Medzhitov & Janeway 2000). Innate immunity also allows time for the acquired immune system to develop an antibody response to the detected pathogen, which may take several days or weeks (Carroll & Forsberg 2007). The cellular components of innate immunity are phagocytic cells such as neutrophils, monocytes and macrophages, which release anti-inflammatory mediators (Carroll & Forsberg 2007). Natural killer cells are also components of innate immunity and serve as the link between innate and acquired immunity (Lynch 2010).

Acquired immunity serves to adapt and build a specific immune response for each antigen that is encountered in the body. This type of immunity is characterised by its production of antibodies that are directed against specific antigens and also acquire the ability of immunologic memory that results in a faster and stronger immune response on subsequent detection of the same pathogen (Carroll & Forsberg 2007). Dendritic and macrophage cells are specialised cells called antigen presenting cells (APCs) and present the detected antigen to a naïve lymphocyte (Lynch 2010) (specialised white blood cells [Carroll & Forsberg 2007]), which evokes a humoral and cellular immune response (Lynch 2010).

The adaptive immune system is comprised of humoral and cellular immunity. Humoral immunity is a part of the adaptive immune system that is evoked by the innate immune system and is known as the antibody-mediated immune response that is responsible for triggering specific B-cells to develop into plasma cells. A large number of antibodies are then secreted by these plasma cells and circulated in the blood and the lymph. Antibodies are a group of proteins called immunoglobulins, whose functions differ (Nauta 2010). Immunoglobulin G (IgG), Immunoglobulin M (IgM) and Immunoglobulin A (IgA) provide defence against viruses, bacteria and toxins, Immunoglobulin E (IgE) offers protection against parasites and allergens and Immunoglobulin D (IgD) has no evident role in defence. The antibodies of the humoral immune response act by attacking and invading the perceived pathogen, binding to it and subsequently marking the pathogen for destruction by cells called phagocytes. Antibodies can be further categorised by those that activate complement serum proteins or those that bind to antigens. Complement serum proteins that are activated by specific antibodies are then able to destroy the pathogen. Antibodies that bind to the antigens are known as neutralising antibodies, and once bound the antigen is no longer able to recognise the host cell, therefore inhibiting the further infection of cells (Nauta 2010; Parham 2014).

Cellular immunity is also known as cell-mediated immunity (CMI) and is mediated primarily by small lymphocytes derived by the thymus – T cells. Two types of T cells exist, the T helper cells and the T killer cells (Nauta 2010). T helper cells play a crucial role in maximising the capabilities of the immune system by activating and directing other immune cells to destroy infected cells or pathogens. A second function of the T helper cells is to stimulate B-cells to secrete antibodies that activate phagocytes which subsequently activate the killer T cells (Nauta 2010). The major function of killer T cells is its ability to recognise the cytotoxicity of cells infected with a virus and destroy these cells, as well as defending the organism against intracellular bacteria. Intracellular bacteria are not detected by the antibodies and macrophages, and therefore, the clearance of infection depends on cytotoxic lymphocytes to eliminate the infected cells. The fact that killer T cells are highly specific with respect to the antigens that they recognise contributes to the uniqueness and effectiveness of the acquired immune response (Nauta 2010; Parham 2014).

Glucocorticoids directly influence the activity of the immune system (Carroll & Forsberg 2007). As previously mentioned, a stressor can be categorised as being acute or chronic. The degree of the perceived stress on the immune system and function may, therefore, be bi-directional. Acute stressors may evoke an immuno-enhancing effect, resulting in the proliferation and differentiation of immune cells, whereas chronic stressors have an opposite effect by evoking an immunosuppressive response (Carroll & Forsberg 2007). The suppression of the immune system is firstly noticed at a cellular level, and as the stress persists, its effects can be examined across the entire immune system (Carroll & Forsberg 2007).

The predominant stressors that result in immunosuppression are transport (Chen et al. 2015) and handling (Trunkfield & Broom 1990). These stressors are seen to involve a complex mixture of unfavourable stimuli that act on the animal and, depending on the nature of methods used, result in lesser or greater effects in stress response. The transport procedure involves handling while loading and unloading, the removal from a familiar to an unfamiliar environment and disruption of social structure due to mixing with unfamiliar animals (Trunkfield & Broom 1990).

Studies conducted to measure cortisol concentrations during the transport procedures have shown an increase in blood cortisol concentrations, resulting in an increased neutrophil to lymphocyte ratio and ultimately causing increased disease susceptibility to bovine respiratory disease (Chen et al. 2015).

The suppression of the immune system may also result in a multifaceted disease complex (Blecha 2000) that arises from viral–bacterial synergy (Aich, Potter & Griebel 2009). When the immune system is impaired due to a chronic stress, the onset of a primary viral infection may increase the animal’s susceptibility to a bacterial infection (Aich et al. 2009). An example of this phenomenon is bovine respiratory disease (Blecha 2000). Cattle whose immune system is already compromised by a viral infection and stress become more susceptible to bacterial pathogens that subsequently invade the bovine respiratory tract resulting in full-blown bovine respiratory disease (Blecha 2000).

Temperament and genetics also influence the animal’s susceptibility to disease (Burdick et al. 2011; Hughes et al. 2013). Studies conducted on steers found that there was an increased negative impact on the acquired immune function in steers that were more temperamental (Burdick et al. 2011). Components of the acquired immune system were affected, resulting in lower lymphocyte proliferation in vitro and decreased *in vivo* vaccine-specific immunoglobulin concentrations (Burdick et al. 2011). Other studies that were conducted on calves showed that more temperamental calves had a decreased response to vaccinations, which is an important part of acquired immunity. The unresponsiveness to vaccines was a result of higher concentrations of plasma glucocorticoids, which have an inhibitory effect on the immune system. Genetics contributes to immune function due to its important role in determining the physical and physiological characteristics of an animal. Animal production, therefore, favours the selection of animals that are genetically less susceptible to disease in order to improve their overall health, performance and productivity (Hughes et al. 2013).

The assessment of disease in animals is of considerable importance when evaluating animal welfare. Animals that are kept in a way in which their immune systems are compromised and are ineffective in combating disease are indicative of management and housing systems that are inadequate and ultimately indicates that the welfare of the animal is at risk (Broom 1991).

## Stress and reproductive success

The failure of an animal to reproduce not only results in the loss of genetic potential but also jeopardises the survival of the entire breed. An animal will make important physiological sacrifices to ensure that it maintains reproductive success, with only the most adverse threats preventing the animal from reproducing (Moberg 1985). There are numerous ways in which stress influences reproduction and involves a number of paracrine, endocrine and neural systems (Von Borell et al. 2007).

Reproductive consequences in dairy cattle are easily examined due to their unique experience of repeated cycles of pregnancy during their lifetime (Mallard et al. 1998). During the transition from pregnancy to motherhood, many physical, metabolic and physiological adjustments have to be made in order to accommodate pregnancy, parturition, as well as the onset of lactation. The exposure of the animal to environmental and management-related changes, including dietary changes, social regrouping, pen moves, encountering the milking parlour (Sepúlveda-Varas et al. 2013) and weaning (Lynch 2010), induces stress in both mother and foetus during gestation (Lay et al. 1997) and in the mother and calf after pregnancy (Mallard et al. 1998).

Foetal development is a critical stage for developmental programming, which is a term that describes the association between environmental challenges that a mother is subjected to during pregnancy and the effect that this has on the developing foetus (Harris & Seckl 2011).

As explained previously, the perception of a stress leads to the release of glucocorticoids due to the activation of the HPA axis (Lay et al. 1997). Foetal development is affected by glucocorticoids due to its interaction with gene expression (Harris & Seckl 2011). The activity of glucocorticoids associated with stress and non-stress activities is regulated by two receptors that differ in affinity for glucocorticoids. Mineralocorticoid receptors have a high affinity for glucocorticoids, whereas glucocorticoid receptors have a low affinity for glucocorticoids. These receptors are present in various target cells that are widely distributed in tissues encountered in circulation (Lynch 2010). Glucocorticoids exert their effect on the developing foetus by binding to these receptors that then subsequently act as transcriptions factors, resulting in the alteration of gene expression. Glucocorticoid receptors are abundantly present in the majority of foetal tissues, including the placenta, in early embryonic development. During early foetal development glucocorticoids aid in the normal development of the maturation of the lungs, correct brain development, remodeling of axons and dendrites as well as affecting cell survival. The expression of mineralocorticoid receptors only appears in later stages of development. Glucocorticoids may have a range of effects on the developing foetus; however, the severity depends on the concentrations that the target tissues or organs or systems are subjected to (Harris & Seckl 2011).

Although chronic stress has an immunosuppressive effect on cattle (Salak-Johnson & McGlone 2007), the exposure of a pregnant cow to mild or acute stressors may be advantageous to the calf (Lay et al. 1997). These advantages arise from the study of plasma cortisol concentrations, which are seen to be elevated in the calf (Lay et al. 1997). When pregnant cattle were exposed to intermittent transport stress during gestation from 60 to 140 days, cortisol concentrations in the calf remained elevated for a longer period of time, resulting in prolonged activation of the HPA axis, and could further result in permanent activation (Lay et al. 1997). The advantage of maintaining high blood cortisol concentrations in the calf may enable it to better cope with mild stress after birth; however, chronic stress may be seen as harmful due to the damaging effects of cortisol on immune function (Lay et al. 1997).

A calf is not only subjected to stress during pregnancy, as the requirement for the calf to respond to homeostatic changes continues after birth due to subjection to environmental and management associated stressors. Calves are born with a functional immune system, which allows for the capability of the calf to respond to certain antigenic stimuli. Due to the fact that maternal antibodies and proteins are not transferred to the calf via the placenta during pregnancy, at birth, the calf experiences an immunological naiveté. To ensure that the calf does not succumb to the infection of pathogens that could increase the risk of survival, it is paramount that the calf receives antibody-rich colostrum, which is produced by the mother (Mallard et al. 1998). The consumption of colostrum is the mechanism by which the passive transfer of maternal antibodies to the neonatal calf is accomplished (Donovan et al. 1986). The colostrum enables the calf to support its own defence mechanisms until they have become fully matured (Mallard et al. 1998).

The first 24 hours after calving are critical for the survival of the calf (Stott et al. 1976). Even though the uptake of colostrum provides the calf with antibodies to respond and survive the effects of pathogens, the calf is hugely at risk to environmental stressors, for instance, heat (Stott et al. 1976). Neonatal calves that are exposed to higher ambient temperatures have a marginally higher body temperatures which in turn increases the concentration of cortisol in the body. Elevated cortisol concentrations in the blood influence the permeability of cells in the small intestine rendering the calf incapable of absorbing macromolecules such as immunoglobulins from colostrum. As a consequence, the transfer of passive immunity from the mother to calf is affected which in turn impairs the calf’s immunity, making it susceptible to disease and increasing the chances of mortality (Stott et al. 1976).

Calves are subjected to further stress at the onset of weaning, which occurs due to the gradual decline in the availability of milk from the mother. The transition of the calf from nutritional and social dependence on the mother to complete independence gives rise to a number of stressors that the calf is subjected to. In cattle production, weaning occurs much earlier than natural weaning, with dairy calves being weaned several hours after birth (Lynch 2010). The weaning of young calves brings about behaviour and nutritional stress, as young calves possess an immature ruminant digestive system that results in the alteration of metabolic and stress-related hormone levels, ultimately affecting immune responsiveness (Pollock et al. 1992).

In light of the above-mentioned factors, life expectancy and reproductive success of the mother and calf is often reduced, which is indicative of the animal battling to cope with its changing environment. The inability of the animal to cope with adverse stimuli suggests that the welfare of the animal may be compromised and ultimately questions reproduction procedures in the cattle production (Broom 1991).

## The hypothalamo-pituitary-adrenocortical axis – significance in assessment of animal welfare in cattle

The term ‘animal welfare’ has arisen in society to convey the ethical concerns about the quality of life that animals experience, particularly those animals that are involved in agriculture (Duncan 2005). Although the term ‘animal welfare’ is not expressed as a scientific concept, scientific methods are employed to identify, interpret and implement the societal concerns surrounding an animal’s quality of life, and therefore, animal welfare has become accepted as a scientific field (Duncan 2005). Without taking for granted that science plays a crucial role in solving animal welfare problems (Duncan 2005), animal welfare must also be considered as a multifaceted issue that is not only comprised of scientific dimensions but also has ethical, economic and political considerations (Carenzi & Verga 2009). Animal welfare must, therefore, be considered on a multi-disciplinary approach that combines researchers from different disciplines within the biological sciences (Carenzi & Verga 2009).

Various groups evaluating the welfare of an animal have different viewpoints depending on their profession (Duncan 2005). For example, a veterinarian’s and a farmer’s biggest concern is the biological functioning of the animal regarding disease, injury, poor growth rates and reproductive problems (Rushen et al. 2007). However, the public are more concerned about the emotions or the affective state of the animal, which entails suffering from unpleasant experiences, pain, fear and hunger (Rushen et al. 2007). These varying viewpoints emphasise the multifaceted dimensions of the evaluation of animal welfare and that when conducting welfare assessments it is important that one keeps all considerations in mind (Duncan 2005).

Although the driving force for the evaluation of animal welfare is the societal concerns of an animal’s feelings, one must remember that feeling is subjective. For example, humans are able to communicate about a certain experience and how that made them feel. It is therefore understood that when humans have an unpleasant experience they usually associate the same feelings with that experience. Because animals communicate in a language that we are not able to understand, it is difficult to assess what each animal feels when it is subjected to a certain experience. The feelings of the animal are poorly defined therefore making them impossible to measure directly. Alternatively, the use of good and poor biological functioning of the animal as an indicator of animal welfare is seen to be more advantageous than the assessment of the animal’s feelings because the variables that are involved are substantive and fairly easy to measure (Duncan 2005).

The evaluation of the HPA axis highlighted in this article provides a scientific approach to the evaluation of the biological functioning of the animal. The HPA axis is activated in response to an endogenous or exogenous stressor in order to help the animal cope with the perceived stress (Lynch 2010). Cortisol is the main product of the activation of the HPA axis and evokes a response from specific target tissues or systems, eventually restoring homeostasis (Mormède et al. 2007). The cortisol levels also fluctuate with different types of stimuli that the animal is subjected to and can, therefore, be directly measured (Mormède et al. 2007). Prolonged stimulation of the HPA axis results in consequences for immune function and reproduction, which are indicators of the biological functioning of the animal (Rushen et al. 2007). Due to the fact that the HPA axis can provide measurable results of the quality of the biological functioning of the animal, its application to the evaluation of animal welfare ensures objectivity (Duncan 2005).

In light of the above, it is imperative that the evaluation of animal welfare incorporates both the biological functioning and the societal concerns of an animal’s feelings because society constantly poses questions regarding the ethicality of an animal’s quality of life in agriculture and science provides the evidence (Duncan 2005).

## Conclusion

It is apparent that numerous stressors arising from both endogenous and exogenous aspects (Lynch 2010) of the cattle production cycle have potentially inhibiting effects on overall productivity and well-being of an animal (Carroll & Forsberg 2007). The HPA axis functions as a coping mechanism to stress and re-adjusts an animal’s homeostatic state by increasing secretion of stress-related hormones (Burdick et al. 2011) and bringing about a behavioural, autonomic, endocrine or immune response (Kumar et al. 2012) to enable the animal to cope with the perceived stress (Lynch 2010). Immune and reproductive function are directly regulated by glucocorticoids (Chen et al. 2015), the final product resulting from the activation of the HPA axis (Lynch 2010). The effects that stress has on the immune function of an animal can be observed by the increase in the occurrence of diseases such as bovine respiratory disease that arise due to stressors associated with transport (Chen et al. 2015). Evaluation of reproductive success showed that stress negatively affects calves throughout their developmental stage, beginning with foetal development and continuing through to adulthood.

Even under the highest quality of management and handling, animals can still be subjected to unfavourable stress (Lay et al. 1997). Emphasis needs to be placed on the fact that good welfare is not achieved by the absence of negative experiences but rather by the higher occurrence of gratifying positive procedures (Lynch 2010). Helping an animal to attain optimum production potential can be desirable as long as there are no indications of poor welfare (Broom 1991). Emphasis should be placed on the fact there is nothing inherently bad about stress unless detrimental effects are observed (Moberg 2000). It is, therefore, imperative that conclusions from scientific studies regarding animal welfare should be made on factual evidence rather than emotive grounds (Broom 1991).

In order to bring about improvements to animal welfare in the cattle production industry, studies should evaluate the preferences of these animals as one needs to know what an animal prefers in order for them to be treated in a humane way (Broom 1991). The impact of stress on animals is too important to be avoided (Moberg 2000) since animals are also sentient beings with the capability of feeling and suffering (Lynch 2010).
